# A Model for Highly Fluctuating Spatio-Temporal Infection Data, with Applications to the COVID Epidemic

**DOI:** 10.3390/ijerph19116669

**Published:** 2022-05-30

**Authors:** Peter Congdon

**Affiliations:** School of Geography, Queen Mary University of London, Mile End Rd., London E1 4NS, UK; p.congdon@qmul.ac.uk

**Keywords:** autoregressive, endemic, epidemic, spillover, regime-switching, spatio-temporal

## Abstract

Spatio-temporal models need to address specific features of spatio-temporal infection data, such as periods of stable infection levels (endemicity), followed by epidemic phases, as well as infection spread from neighbouring areas. In this paper, we consider a mixture-link model for infection counts that allows alternation between epidemic phases (possibly multiple) and stable endemicity, with higher AR1 coefficients in epidemic phases. This is a form of regime-switching, allowing for non-stationarity in infection levels. We adopt a generalised Poisson model appropriate to the infection count data and avoid transformations (e.g., differencing) to alternative metrics, which have been adopted in many studies. We allow for neighbourhood spillover in infection, which is also governed by adaptive regime-switching. Compared to existing models, the observational (in-sample) model is expected to better reflect the balance between epidemic and endemic tendencies, and short-term extrapolations are likely to be improved. Two case study applications involve COVID area-time data, one for 32 London boroughs (and 96 weeks) since the start of the COVID epidemic, the other for a shorter time span focusing on the epidemic phase in 144 areas of Southeast England associated with the Alpha variant. In both applications, the proposed methods produce a better in-sample fit and out-of-sample short term predictions. The spatial dynamic implications are highlighted in the case studies.

## 1. Introduction

The context for modelling spatio-temporal infectious disease data is set by three major considerations. The first is the extent of instability in the data, with stable infection levels characterising endemic infections, but instability in infections that have epidemic phases. The second is the geographic variation in the infection trajectories, for example, some areas experienced earlier epidemic upturns. The third is the infection spillover from adjacent areas (when the spatial context involves a lattice framework such as administrative areas).

Many models of infectious data focus primarily on modelling and forecasting a single epidemic and its components (i.e., modelling the exponential ascent and subsequent descent). One widespread approach uses phenomenological models [1] and is usually based on national infection counts—these are typically fitted using nonlinear least squares with normality assumed for errors. These models are difficult to extend to multiphase epidemic data, which is when the observation span includes multiple epidemics [2,3]. However, as has become apparent with the COVID outbreak, there is a need for modelling multiphase epidemic data with intervening phases of relatively low infection.

Compartmental models [4] represent infectious disease in terms of separate compartments, with epidemic evolution via differential equations. They rely on assumed or estimated parameters to estimate an epidemic curve, but typically cannot explicitly model endemic and epidemic dynamics, especially for multiple waves [5].

Some models, typically also applied to national infection data, adopt theaAutoregressive integrated moving average (ARIMA) strategy [6,7,8]. These involve preliminary differencing or transformation of the data in order to achieve stationarity and also assume normal errors. Thus, essential features of the data are lost in the analysis.

It is argued in the research here that greater flexibility is provided if nonstationarity is explicitly present in the model and not “differenced away” by preliminary and often complex data manipulation. In explicitly allowing for nonstationarity, the model developed below provides an indicator of which phase or “regime” is predominant at a particular point in the infection time series. This is a form of regime-switching or alternation. Since this model typically reflects better the balance between epidemic and endemic tendencies than existing models, the in-sample fit is expected to improve and short-term extrapolations will also generally produce more accurate forecasts—this is demonstrated in two applied case studies.

Regime-switching has been used in other studies [9,10], though here it is implemented with a smooth mechanism governing regime alternation, rather than discrete switching of regimes. Discrete state switching applies to models assuming a latent Markov chain approach such as [11]. Studies also assume normal outcomes based on differencing population rates in successive time units. This assumption might become problematic for small infection counts when the areas have small populations.

Moreover, accounting for spatial differentiation in trends and epidemic upturn timing is important as well as modelling the multiphase aspects. There are so far few models that have attempted to explicitly model multiphase infection data for multiple areas. In a spatially disaggregated situation, infection counts during particular periods (e.g., between epidemic phases) may be small, and conventional approaches (e.g., transforming or differencing data converted to population rates and also assuming normality) are inappropriate. The models used here are appropriate to the form of the space–time count data. The models used are part of a broader “disease mapping” approach appropriate to count data and applicable to often small area infection counts, particularly in endemic phases [12,13].

Specifically, we develop here a model appropriate to area-specific multiphase count data, assuming Poisson sampling, albeit allowing for overdispersion in the form of a negative binomial. The approach provides a spatio-temporal regime-switching model adapted to small-area disease counts. As well as this feature, the model here includes disease spillover effects between neighbouring areas, a feature not present in many spatiotemporal infection models. The model outputs supply a wide range of information about infectious disease spread and waning, for example, parametric indicators of the relative balance between epidemicity and endemicity in each area and spillover infections from neighbouring areas. These indicators are not available in other models applied to area–time infection counts.

The model here has the benefit (unlike approaches based on ARIMA strategies) of avoiding the need to achieve stationarity by differencing and transformation. The analysis is done on a natural and interpretable scale, namely the infection counts [5].

The output of the model proposed here provides important details regarding the spatial dynamics of epidemics. It provides details about differential epidemic trajectories between areas, for example, in terms of where early epidemic upturns (or early downturns from epidemic peaks) are concentrated. The model also allows area-specific one-step ahead forecasts of epidemic counts, which are important for policymakers concerned with developing localised strategies for epidemic containment.

## 2. Relevant Literature

There have been a considerable number of spatio-temporal studies of disease patterns, generally adopting a Bayesian perspective [12,14]. Spatio-temporal models for infection counts [15,16] are a particular sub-theme. These incorporate the themes of the broader disease mapping literature, such as the gains through borrowing strength and the need to reflect spatial correlation in disease; for example, see Andrews et al. [17] on spatial clustering in COVID rates. Space–time models also need to incorporate the spatial diffusion or spillover related to behaviours such as commuting [18,19]. It is also especially useful in policy terms to be able to extrapolate the infectious disease evolution beyond the observation span, as illustrated in some studies of the COVID epidemic [20,21,22,23].

Low-order autoregression is a feature of several recent spatio-temporal studies of infection data. For example, Paul and Held [24] and Shand et al. [25] adopt first-order autoregression (AR1) models, where autoregressive coefficients on counts or rates in the previous period are spatially varying. The model of Paul and Held includes a spatial lag on infection counts in adjacent areas that allows for neighbourhood spillover effects in infection; related approaches are considered by Martines et al. [26] and Griffith and Li [27]. In infections spread by human contact, it is implausible that higher counts or rates in one period generate smaller infection levels in the next period, and so a positive constraint on the AR1 coefficient is justified. Stationarity may also be assumed [25,28] with an AR1 coefficient under 1, but the analysis below argues that flexibility to pronounced epidemic fluctuations in infection counts is likely to be gained by allowing nonstationarity. Nonstationarity is an option in the Bayesian analysis of AR1 models [29,30].

A particular feature of epidemic time series is that a period of relatively stable infection levels (which can be viewed as an endemic phase) is followed by a sudden sharp phase of increasing infection levels. After the epidemic peak, there is a period of descending rates and a return to stability. Hence, it is argued here that greater flexibility and improved prediction will follow if the autoregressive scheme is allowed to adapt to these pronounced fluctuations with temporary departures from stationarity but returning to stationarity as rates descend and infections resume endemic levels.

In this paper, we consider a mixture-link model for infection counts that allows adaptivity to both explosive phases and to stable endemicity, with higher AR1 coefficients in epidemic phases. This is a form of regime-switching. We also allow for neighbourhood spillover, which is also governed by the adaptive switching mechanism.

A Bayesian estimation approach is used. Assuming an identity link in the count regression, AR1 coefficients exceeding 1 reproduce sharply increasing infection levels during an explosive phase, whereas AR1 coefficients under 1 are associated with stability. Compared to existing models such as [24,25], the observational model will then better reflect the balance between epidemic and endemic tendencies and so provide a better fit, and short-term extrapolations will also generally produce more accurate forecasts.

## 3. Case Studies

We consider two case study applications involving area–time data for COVID-19 infection counts. These provide differing spatial perspectives and involve different variants underlying the epidemic peaks. The use of two studies provides stronger evidence that a better fit due to the proposed models is not due to the particularities of one dataset alone.

The first case study considers the 32 London boroughs and focuses on the sudden growth in COVID infections due to the Omicron variant at the end of 2021. The link-mixture approach is applied to data from the start of the epidemic in March 2020 through to early 2022 for the London boroughs (96 weeks of infection totals) and shows better fit and improved short-term forecasts over models without regime alternation.

The second case study considers data for a much wider regional framework, namely Southeast England consisting of 144 areas as opposed to 32 areas in the first case study. This study considers a shorter time span, the weeks up to and including the peak of infections due to the Alpha variant at the end of 2020.

## 4. Methods

### 4.1. Autoregression for Area–Time Infection Counts

Consider area–time infection count data $y_{it}$ for areas i=1,…,N and times 1,…,T, and assume these are negative binomial (NB), $y_{it}∼NB\left(\mu_{it},\Psi \right).$ The negative binomial model is a generalisation of the Poisson density and appropriate to count data which may be overdispersed relative to the Poisson, for example, COVID infection counts in the endemic phase may even include zeroes, whereas in the epidemic phase much higher counts occur. Hence, the data are overdispersed with variance exceeding the mean. The NB parameterisation is

$$p\left(y|\mu ,\Omega \right)=\frac{\left(y+\Psi -1\right)!}{y!\left(\Psi -1\right)!}{\left(\frac{\mu }{\mu +\Psi }\right)}^{y}{\left(\frac{\Psi }{\mu +\Psi }\right)}^{\Psi }.$$

Assume an AR1 model on previous infection counts in the same area. Additionally, effects of predictors $X_{it},$ and unobserved area effects $u_{i}$, may be represented by a term

$$\eta_{it}=X_{it}\beta +u_{i}.$$

Then, for a basic model, conditioning on the first period’s data, we adopt an identity link

(1) $$\mu_{it}=\rho_{i}y_{i,t-1}+\exp(\eta_{it}),\;\;\;\;\;\;\;\;\;\;t=2,\ldots ,T$$

providing positivity in $\rho_{i}$ is ensured.

Including lags on infection counts in nearby areas reflects infection spillover due, for example, to social interactions between residents in neighbouring areas, or to cross-boundary commuting [19]. Let $h_{ij}$ measure spatial interactions between areas i and j, and $w_{ij}=h_{ij}/\sum_{j}h_{ij}$ be row standardised spatial weights, with $\sum_{j}w_{ij}=1$. Then spatial spillover, also with lag 1, can be represented [24] by adding a spatially averaged term $\lambda_{i}\sum_{j}w_{ij}y_{j,t-1}$ to the above basic model. Then, one has

(2) $$\mu_{it}=\rho_{i}y_{i,t-1}+\lambda_{i}\sum_{j}w_{ij}y_{j,t-1}+\exp(\eta_{i}),$$

providing positivity in $\rho_{i}$ and $\lambda_{i}$ is ensured.

Assuming that $\rho_{i}$ and $\lambda_{i}$ are positive is justified epidemiologically, since—for infections spread by human contact or interaction—higher current totals of infectees in an area $y_{i,t-1},$ or its vicinity, $\sum_{j}w_{ij}y_{j,t-1},$ are expected to cause higher future infections. A negative effect of existing infection levels on future infections is therefore implausible.

### 4.2. Link Specification

One then requires an appropriate link function relating $\rho_{i}$ and $\lambda_{i}$ to relevant parameters. For example, assume spatially correlated conditional autoregressive random effects $f_{1i}$ and $f_{2i}$ [31] involved in predicting $\rho_{i}$ and $\lambda_{i}$, and assume these are zero-centred. The study ([24], p. 1121) adopts a log link by default so that with intercept terms $\alpha_{1}$ and $\alpha_{2}$, one has

(3) $$log\left(\rho_{i}\right)=\alpha_{1}+f_{1i},\;log\left(\lambda_{i}\right)=\alpha_{2}+f_{2i}.$$

A log link allows for explosive effects ($\rho_{i}$ and/or $\lambda_{i}$ exceeding 1) but does not necessarily select explosive behaviour. If most of the epidemic series consists of stable infection levels (endemicity), then the estimated $\rho_{i}$ and $\lambda_{i}$ are likely to be below 1.

For infectious diseases with endemic recurrence now predominant, such as HIV in developed nations, a stationary autoregressive effect may be seen as appropriate. See, for example, Shand et al. [25] who consider time variations in HIV in US counties. For an AR1 model on lagged infections, this implies a logit link with $\rho_{i}$ and $\lambda_{i}$ are constrained between 0 and 1. Thus, with the same overall model (2), and spatial effects $f_{3i}$ and $f_{4i},$ one has

(4) $$logit\left(\rho_{i}\right)=\kappa_{1}+f_{3i},\;logit\left(\lambda_{i}\right)=\kappa_{2}+f_{4i},$$

Neither of the models in [24] or [25] includes a regime-switching mechanism.

### 4.3. Choosing between Epidemic or Endemic Phases (Link Mixing)

However, for infectious diseases such as COVID, switching between epidemic and endemic phases is relevant to the effective modelling of wide fluctuations. Hence, a logit link is relevant when infections are at a low and/or stable level, whereas a log link allowing $\rho_{i}>1$ and $\lambda_{i}>1,$ is more flexible in periods with explosive growth in infections (e.g., due to a new virus or new variants of that virus). An example is the rapid increase in COVID infections linked to the emergence of the Omicron variant, as considered in the first case study.

Here, we consider a mixture model facilitating time-variations in which link is predominant so reflecting the current infection phase. Other forms of mixing between links have been considered in other types of applications (not involving infectious disease counts) or extra parameters introduced into modelling links. For example, Lang [32] considers a mixture of the canonical symmetric logistic link and one or more asymmetric forms in modelling ordinal and binary outcomes, whereas Czado and Raftery [33] consider right and/or left tail modifications to standard links.

Here, we consider a situation not researched before (as far as the authors are aware), namely choosing between log and logit links. Thus, for weights $\omega_{t}$ between 0 and 1, it is here proposed that

(5) $$\rho_{it}=\omega_{t}\exp(\alpha_{1}+g_{1i})+\left(1-\omega_{t}\right)\frac{\exp(\kappa_{1}+g_{1i})}{1+\exp(\kappa_{1}+g_{1i})},\;\lambda_{it}=\omega_{t}\exp(\alpha_{2}+g_{2i})+\left(1-\omega_{t}\right)\frac{\exp(\kappa_{2}+g_{2i})}{1+\exp(\kappa_{2}+g_{2i})},$$

where $\rho_{it}$ and $\lambda_{it}$ now vary by area *i* and time *t*, and $g_{1i}$ and $g_{2i}$ are spatially correlated conditional autoregressive random effects. The $\omega_{t}$ are in effect measuring stability or instability in infection rates and so are taken as common to both own area and the neighbouring area lags, $\rho_{it}$ and $\lambda_{it}$, respectively. For $\omega_{t}$ high and approaching 1, infections are typically rapidly increasing, whereas for low $\omega_{t},$ stable endemicity is indicated. Low $\omega_{t}$ may also be better for characterizing the descent phase after epidemic peaks.

There is no reason why spatial patterning in autocorrelation should be the same in epidemic or endemic phases, so a variation on the preceding model allows for differing spatial effects between phases, namely

(6) $$\rho_{it}=\omega_{t}\exp(\alpha_{1}+g_{1i})+\left(1-\omega_{t}\right)\;\frac{\exp(\kappa_{1}+g_{3i})}{1+\exp(\kappa_{1}+g_{3i})},\;\lambda_{it}=\omega_{t}\exp(\alpha_{2}+g_{2i})+\left(1-\omega_{t}\right)\frac{\exp(\kappa_{2}+g_{4i})}{1+\exp(\kappa_{2}+g_{4i})}.$$

### 4.4. Alternation Mechanism

The $\omega_{t}$ in (5) and (6) are modelled as time-specific beta variables

(7) $$\omega_{t}∼Beta\left(q_{1t},q_{2t}\right),$$

where $q_{1t}$ and $q_{2t}$ are positive parameters. The $\omega_{t}$ are between 0 and 1 and so provide smooth alternation between endemic and epidemic phases. The specification in (7) provides a simple approach (and parsimonious in parameterisation) to regime alternation in a situation with multiple epidemics. More elaborate smooth transition schemes have been used [34] but are typically framed for the case of a single transition between regimes, whereas multiple transitions are involved in multiphase epidemics.

In contrast to smooth alternation, discrete switching mechanisms using binary switching indicators as in Markov chain switching [10,11] imply the unequivocal distinction between endemic and epidemic phases with, for example, one week classified as endemic and the next week as epidemic. These may be heavily parameterised, for example, the model in [11] has latent binary indicators *Z_its_* according to area, time, and season (in a model for influenza cases).

Relevant covariates if available, possibly time-lagged, may be used in predicting the $\omega_{t}$ via beta regression. Regression for the mixing variables can be handled by the parameterisation

$$\omega_{t}∼Beta\left(M_{t}\pi_{t},M_{t}\left[1-\pi_{t}\right]\right),$$

with $\pi_{t}$ being probability parameters (explained by the covariates), and $M_{t}$ positive parameters.

### 4.5. Other Model Features

The models in (2) may be extended to include time and area–time varying effects, such as seasonal effects, or unobserved area–time random effects $\delta_{it}$. These represent local trends not fully captured by autoregressive effects on lagged infection levels. Thus, for representations (3) and (4), one has

(8) $$\mu_{it}=\rho_{i}y_{i,t-1}+\lambda_{i}\sum_{j}w_{ij}y_{j,t-1}+\exp(\eta_{i}+\delta_{it}),$$

whereas for representations (5) and (6), one has

(9) $$\mu_{it}=\rho_{it}y_{i,t-1}+\lambda_{it}\sum_{j}w_{ij}y_{j,t-1}+\exp(\eta_{i}+\delta_{it}).$$

### 4.6. Summary Epidemic Indicators

Under both (5) and (6), focusing on area variations in $\rho_{it}$ and $\lambda_{it}$ during periods with explosive growth will indicate which areas have been more subject to such growth. These indicators will tend to be highest in the periods just before the epidemic peak when cases are growing fastest.

A number of summary epidemic indicators can be derived. Thus, the summary coefficients ${\bar{\rho }}_{t}$ and ${\bar{\lambda }}_{t}$, obtained by averaging $\rho_{it}$ and $\lambda_{it}$ over areas, give an overall impression of infection growth or endemic phases. The $\rho_{it}$ and $\lambda_{it}$ can also be compared to the threshold of 1 to give a probability indication of explosive growth in different areas. Thus, define indicators

$$r_{it}^{x}=I(\rho_{it}>1),\;l_{it}^{x}=I(\lambda_{it}>1),$$

from which area–time exceedance probabilities can be estimated. Also, the sums $R_{t}^{x}=\sum_{i}r_{it}^{x}$ and $L_{t}^{x}=\sum_{i}l_{it}^{x}$ show total areas with explosive infection growth in each period.

To assess effectiveness of spatial predictions (i.e., area-specific predictions), one may compare observed growth rates in cases *y_i_*_,*t*+1_/*y_i_*_,*t*_ with modelled growth rates *μ_i,t_*_+1_*/μ_i,t_* This comparison is particularly relevant in epidemic phases or in assessing short-term forecasts.

### 4.7. Model Specification

The forms (8) and (9) are adopted in the case studies below. The spatial effects $\left(f_{1i},f_{2i}\right),\;\left(f_{3i},f_{4i}\right)$ and $\left(g_{1i},g_{2i},g_{3i},g_{4i}\right)$ involved in defining the autoregression coefficients are taken to follow the conditional autoregressive (CAR) scheme of [31]. It is assumed that

$$\eta_{it}=X_{it}\beta +u_{i},$$

where $u_{i}$ are mean-centred CAR spatial effects as in [31]. It is assumed that the area–time effects $\delta_{it}$ follow a first-order random walk $\delta_{it}∼N\left(\delta_{i,t-1},\sigma_{\delta }^{2}\right),$ with initial conditions $\delta_{i1}$ taken as fixed effects, $\delta_{i1}∼N\left(0,1\right)$. For identification, an intercept is omitted from $X_{it}\beta$ and covariates are centred. A single covariate is used in both case studies; the mid-2020 population estimates are divided by 100,000.

Gamma priors with shape one and rate 0.01 are adopted on inverse variance parameters, the parameters $\left\{q_{1t},q_{2t}\right\}$, and on the negative binomial overdispersion parameter Ω. Normal N(0,100) priors are assumed on fixed effects $\left\{\alpha_{1},\alpha_{2},\kappa_{1},\kappa_{2},\beta_{1}\right\}.$ We consider one-step ahead predictions. The predictive means are taken as

$$\mu_{i,T+1}=\rho_{iT}y_{i,T}+\lambda_{iT}\sum_{j}w_{ij}y_{j,T}+\exp(\eta_{it}+\delta_{i,T+1}),$$

and include the updated value $\delta_{i,T+1}∼N\left(\delta_{iT},\sigma_{\delta }^{2}\right)$.

## 5. Analysis and Estimation

We apply the link-mixture models specified in Equations (5) and (6), and the mean as in (9), these constituting models 3 and 4, respectively. Two simpler options are the log link as in (3), constituting model 1, and the other (as model 2) is the logit link as in (4). Models 1 to 4 are denoted M1, M2, M3, and M4, respectively. Bayesian estimation is adopted and implemented via the BUGS program [35]. Two chains of 20,000 iterations are taken with inferences from the last 10,000 and convergence checks as in [36].

Fit is measured by the widely applicable information criterion (WAIC) [37]. The WAIC is a measure of goodness of fit with a penalty for complexity (more complex models receive a greater penalty). Lower values of the WAIC indicate a better fit. The advantages of the WAIC over other fit measures used in Bayesian inference are discussed by Lambert [38]. The performance of predictions $P(y_{rep,it}|y_{it})\;$=$\;\int P\left(y_{rep,it}|\theta \right)P(y_{it}|\theta )d\theta$ (where θ denotes all parameters) is measured by the Dawid–Sebastiani score (DSS) and by the ranked probability score (RPS) [39]. These two criteria are explicitly designed to assess the predictive success of models for count time series and are now incorporated in the R software package (R Foundation for Statistical Computing, Vienna, Austria) [40]. Both these criteria are lower for better fitting models.

Let $Y_{t}$ denote region-wide totals at period t (i.e., total infections for all areas combined). Assume the models are fitted to T time periods with period T+1 as the holdout. One-step ahead predictions for T+1 are assessed by whether these predictions include actual infection counts at T+1 and by the RPS for one-step ahead predictions.

Code and data are provided as Supplementary Materials.

## 6. Case Study 1: London Boroughs, 32 Areas, 96 Weeks

The data for the first study consisted of weekly totals of new COVID cases in the 32 boroughs of London. The time span considered starts (*t* = 1) at the week ending Sunday 8 March 2020, with a final observation (*t* = 96) for the week ending Sunday 2 January 2022.

**Figure 1:**
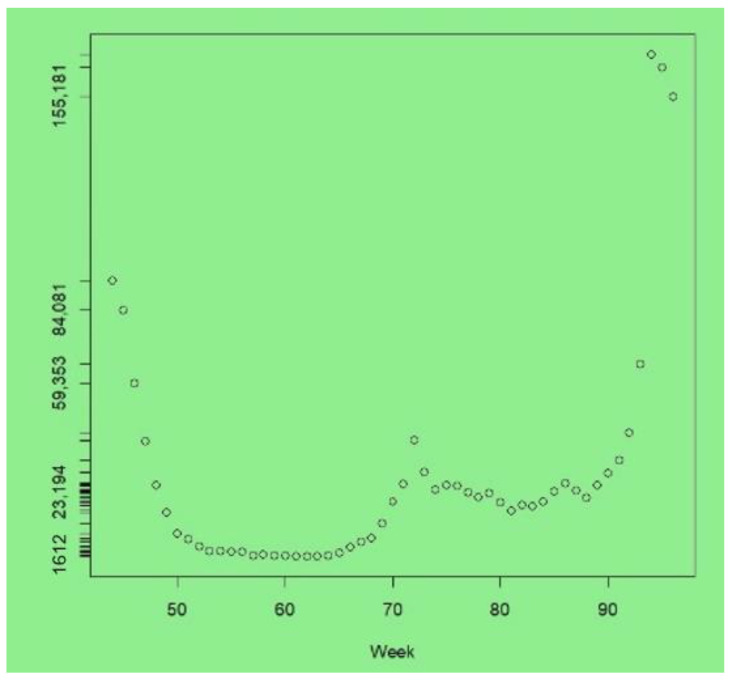
COVID cases, London, weeks 44–96.

The upturn due to the new Omicron variant is apparent in the last few weeks of the series. The peak infections were at week 94 (with 169,322 cases, compared to 65,771 in week 93), after which a downturn started, with 155,181 cases at week 96. At the peak of the London Omicron wave, the UK Office of National Statistics estimated that around 8.8% of Londoners had COVID-19 ([41], Table 1e). In contrast, between weeks 1 and 30, most weeks recorded under 5000 new cases across London, and weeks 52–66 had under 5000 new cases—see Figure 1, which plots cases from weeks 44 to 96.

We take weeks 1–95 as the observed data, with week 96 as the holdout. There were 155181 cases in that week as infection levels due to Omicron started to tail off from the peak in week 94. Table 1 compares the four models in terms of fit to the data and prediction accuracy within the observed span. Table 1 also compares their out-of-sample predictions to week 96.

Regarding fit to the observed data, the WAIC, RPS, and DSS criteria are all lower for the link-choice models (models 3 and 4) than for the default log and logit link models (models 1 and 2 in Equations (3) and (4), respectively).

**Figure 2:**
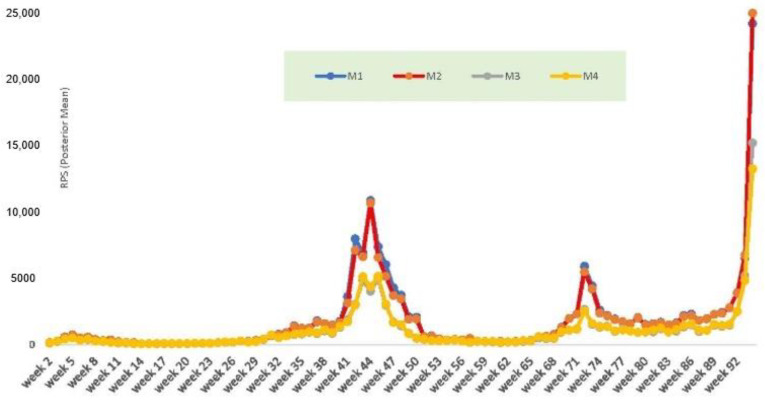
Ranked probability score by week; models compared.

Figure 2 plots out one of the fit measures, the RPS, by week. It shows worse predictions under models 1 and 2 (M1 and M2 are the red and blue lines in Figure 2). Models M1 and M2 show worse fit in an upturn due to the Alpha variant, which produced a peak infection count of 93798 for the week ending 3 January 2021 (week 44 of the series).

Models 3 and 4 also have greater accuracy in one-step ahead prediction in terms of the coverage of the 95% predictive interval for $Y_{rep,T+1}$ of the actual value and the RPS for week T+1. The 95% predictive interval for *T* + 1 under model 4 is (154,018, 175,048) including the true value of 155,181, and the one-step ahead RPS is 42,923. The predictive interval under model 3 also includes the true value. In contrast, models M1 and M2 over-predict $Y_{T+1}$, their 95% predictive intervals for $Y_{rep,T+1}$ do not include the actual value, and the RPS measures of predictive fit are much worse.

The posterior means $\rho_{i}$ under M1 (which are time constant) vary from 0.13 to 0.25, whereas the mean $\lambda_{i}$ varies from 0.19 to 0.72. There is therefore no indication in which areas epidemic growth occurred. In contrast, under models 3 and 4, the $\rho_{it}$ and $\lambda_{it}$ parameters will exceed 1 in weeks with a very high growth in cases.

From the latter parameters (only available under models M3 or M4), one may identify the upturn weeks in which areas have epidemic growth. Table 2, accordingly, shows the 20 weeks with the highest values of $R_{t}^{x}$ under M4. In a few weeks (such as weeks 2 and 94), all 32 boroughs have nonstationary growth, but Table 2 shows that such growth is concentrated in a relatively few weeks in the observation span of 95 weeks.

**Figure 3:**
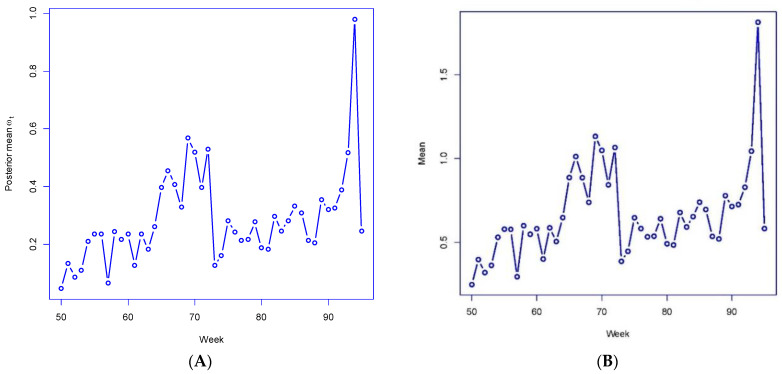
(**A**) posterior mean omega, London boroughs, (**B**) posterior mean ${\bar{\rho }}_{t}$.

Figure 3A,B plot out the posterior mean $\omega_{t}$ under M4 for weeks 50–95 and the averages ${\bar{\rho }}_{t}$ of the $\rho_{it}.$ For these weeks, the $\omega_{t}$ and ${\bar{\rho }}_{t}$ correlate highly (over 0.99) with actual growth rates in London-wide total cases $Y_{t}/Y_{t-1},$ emphasizing how well the parameters reproduce the actual infection data. For 6 of these 45 weeks, the London-wide ${\bar{\rho }}_{t}$ under M4 has a posterior mean exceeding 1 (i.e., rapid case growth in epidemic periods), with the highest ${\bar{\rho }}_{t}$ being 1.81 for week 94. These results confirm the utility of the link-mixture mechanism in reproducing actual infection count fluctuations.

### Spatial Dynamics

**Figure 4:**
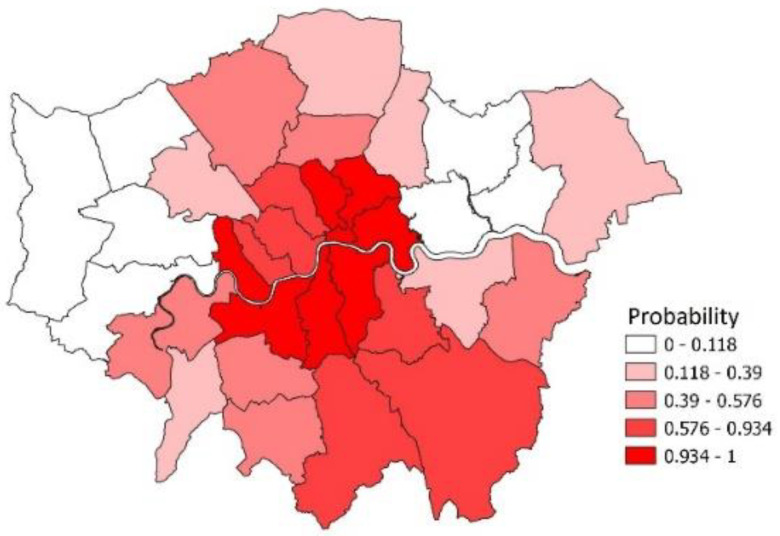
Probabilities of epidemic growth, London Boroughs, Week 93.

The course of infection in particular areas is a major concern. Figure 4 maps out boroughs according to the probabilities $r_{it}^{x}$ in week *t* = 93 (the week preceding the Omicron infection peak in London at week *t* = 94), where $r_{it}^{x}$ are the probabilities of epidemic growth in different areas at particular time points. A spatial concentration of epidemic growth is especially apparent in Southeast London, with the moran.test facility in R yielding a Moran I spatial coefficient of 0.43 with *p*-value under 0.0001.

The predicted area-specific changes in cases between weeks 93 and 94 (when the omicron related epidemic peaked) correlate closely, confirming the model’s utility in representing spatial dynamics. Thus, the correlation between *μ_i_*_,94_*/μ_i_*_,93_ and *y_i_*_,94_/*y_i_*_,93_ over the 32 areas is 0.79. Similarly, in the short-term predictions to week 96, the correlation between *μ_i_*_,96_*/μ_i_*_,95_ and *y_i_*_,96_/*y_i_*_,95_ over the 32 areas is 0.90.

Other spatial aspects of the model, such as the assumed spatial correlation in unobserved area effects $u_{i}$ and the spatial correlation in the autoregressive parameter random effects, are confirmed. For the $g_{1i}\;\mathrm{and}\;g_{2i}$ in the M3 model, we obtain Moran coefficients of 0.33 and 0.07 with respective 95% predictive intervals (0.18, 0.48) and (−0.01, 0.18). So, spatial correlation is stronger in determining the *ρ_i_* than the *λ_i_*. The Moran for the spatial CAR effects $u_{i}$ is 0.44 with a 95% interval (0.06, 0.77).

## 7. Case Study 2: Southeast England, 144 Areas, 20 Weeks

The data for this study relates to the broader southeast of England, encompassing 144 local authority areas in three standard regions (London, East, and Southeast). The time span consists of 21 weeks from the week ending 9 August 2020 through to the week ending 27 December 2020. This period includes a peak in cases related especially to the Alpha variant, namely week 21 with 210,099 cases, whereas in weeks 1–17 there were under 50,000 cases per week. We consider observations for the first 20 weeks, with week 21 held out from estimation. We compare the models in terms of their fit to the observed data (weeks 1–20) and one-step ahead predictions to week 21 when cases peaked.

Table 3 shows, as for the London study, that models 3 and 4 provide a better fit and predictions to the observed data. Table 4 shows the RPS by week for the four models. Models M1 and M2 have a worse predictive fit in weeks with rapid shifts in case numbers (large increases or falls, as in weeks 19 and 15).

**Figure 5:**
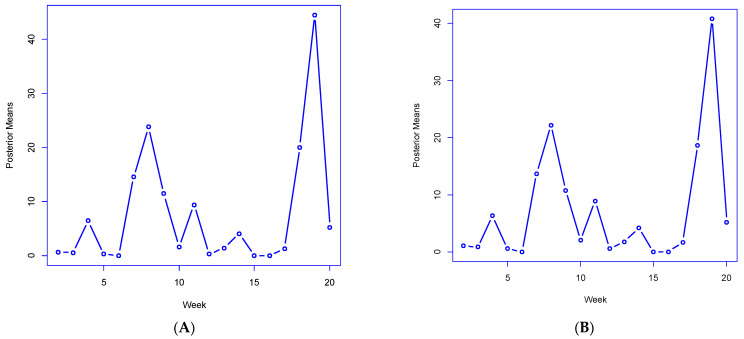
(**A**) Average number of own area slopes exceeding 1, $R_{t}^{x}$. (**B**) Average number of spatial lag slopes exceeding 1, $L_{t}^{x}$.

As to tracking extreme increases associated with the Alpha variant, Figure 5A,B plot the M4 posterior means by a period of the statistics $R_{t}^{x}$ and $L_{t}^{x},$ the total number of areas with slopes $\rho_{it}$ or $\lambda_{it}$ exceeding 1 (consistent with epidemic growth). These both peak in week 19, at 44.5 and 40.8, respectively (out of a total of 144 areas), implying that the sharp growth in cases is from both local transmission and broader geographic diffusion. These statistics closely correlate (0.86 and 0.87, respectively) with observed growth ratios $Y_{t}/Y_{t-1},$ confirming the utility of derived model indicators in reproducing actual infection dynamics.

Models 3 and 4 also have better predictive out-of-sample performance for week 21 than M1 and M2. For example, the 95% predictive interval for the region-wide total at T = 21, namely $Y_{rep,T+1}$ under model 4 is (194,169, 214,482) comfortably including the actual value of 210,099. In contrast, models M1 and M2 tend to underpredict the future value.

As for the previous case study, spatial dynamics are of major importance. We find that all the random effects relevant in the best-fitting M4 are confirmed as spatially correlated: the Moran I for *u_i_* $g_{1i}$, $g_{2i},\;g_{3i},$ and $g_{4i}$ are, respectively, (with 95% intervals) 0.26 (0.06, 0.47), 0.32 (0.13,0.62), 0.45 (0.24, 0.71), 0.51 (0.29,0.74), and 0.44 (0.24,0.64).

**Figure 6:**
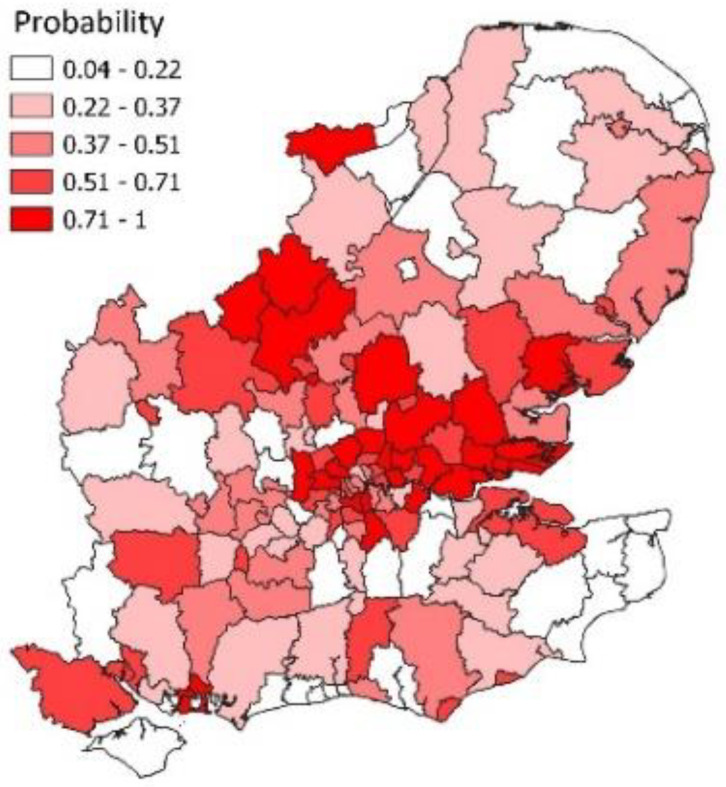
Probabilities of epidemic growth, Greater Southeast, week 19.

Figure 6 shows the spatial pattern of epidemic probabilities in week 19 when there was a near doubling of cases. As for Figure 4, there is spatial clustering in infection growth with a Moran statistic of 0.44 (with highly significant *p*-value). Such clustering supports features of the model such as allowing for neighbourhood infection spillover.

## 8. Discussion

The literature on epidemic modelling has paid little explicit attention to methods for spatially disaggregated infection data in a situation of multiple epidemic phases with intervening spells of endemic infection. Many studies involve a single region or nation [9,10]. The present paper has proposed a methodology for spatially disaggregated infection counts including both regime alternation in situations with multiple epidemic phases and neighbourhood spillover in infection. The advantages of the methods presented here have been shown in two case studies, distinct in the epidemic virus considered and in their spatial framework.

Existing models for area–time infection counts mostly include no mechanism to distinguish epidemic from endemic phases, and hence short-term forecasts using them will tend to be less accurate than the methods proposed here. Approaches based on phenomenological models (e.g., logistic curves) or compartmental models, are difficult to adapt to multiple epidemic phases or to spatially disaggregated data, whereas ARIMA models generally use data differencing and transformation rather than analyse the data as they are. They are also difficult to extend to multiple areas, i.e., to a spatio-temporal situation.

In contrast, the method proposed in this paper adapts to nonstationarity in cases and to spatially disaggregated data. For infection count time series with epidemic phases, stationarity is a restrictive assumption and allowing nonstationarity is appropriate [42]. The model proposed here includes novel features such as a mechanism to represent epidemic against endemic phases, both in the aggregate (region-wide) and for individual areas and applying both to local infection spread and infection diffusion from neighbouring areas. The model can be seen as a spatio-temporal regime-switching model. We avoid transformation used in some spatio-temporal infection models, e.g., [43], and retain counts as a natural metric [5].

A number of diagnostic statistics are presented (with relevance to interpreting the time course of an infectious disease) and shown in the case studies to closely reproduce actual infection trends. An example is the match between the diagnostics in Figure 3A,B with actual growth in London cases, with a similar close correlation in the Greater Southeast case study.

Another result of these novel features is that the link-mixture model produces a better representation of spatial dynamics and improved short-term forecasts. Forecasts of infection change at area-specific levels (such as the 32 areas in the first case study) correlate positively with actual changes in cases.

The case studies in this paper have used relatively large areas (e.g., averaging 170 thousand in the London case study), but the approach used remains appropriate for smaller neighbourhoods (e.g., areas with around 10,000 population), where small infection counts are likely to be involved. A generalised Poisson model with Bayesian smoothing mechanisms to borrow strength remains suitable at lower spatial disaggregation. The approach of this paper may also be used with other outcomes, possibly usually less frequent than cases, such as infection-related deaths.

Possible extensions or variations of the approach proposed above may be considered. One is to make the mixing variables *ω_t_* area-specific, namely *ω_it_*, though at the expense of extra parameterisation and possibly weakened identification. Another, as suggested above, is to introduce covariates to explain the disease course. This could be done via the *X_it_* in *η_it_*, or in a beta regression for the *ω_t_*. Covariates might be infection-related such as the proportions of infections due to a new variant. Intervention or environmental variables may also be included in this regression. For example, there is increasing evidence of links between COVID infection and weather conditions [44].

Another potential extension is to multiple outcomes (e.g., cases and hospitalizations), for example, using multivariate CAR spatial effects in (5) and (6). This type of model might include the time-lagged dependence of hospitalisations on cases.

The methodology proposed here may have application beyond infectious disease counts, particularly to longitudinal spatial count data involving considerable time fluctuations. In spatial applications, it is relevant when positive feedback from neighbouring locations is anticipated on substantive grounds [45]. Possible examples include urban crime [46] and spatial innovation diffusion [47].

## 9. Conclusions

Many epidemic time series—COVID being a current example—show periods of relatively stable infection levels (characterisable as endemicity), followed by phases of rapidly increasing infection levels. After the epidemic peaks, there is a period of descending rates and a return to stability. Hence some mechanism is needed to alternate repeatedly between epidemic and endemic phases or “regimes”.

The regime-alternation specification used in this paper is relatively simple, applicable both to local infection spread and spread from neighbouring areas and can be adapted to multiple epidemic phases. It is parsimonious in parameter terms, whereas considerably heavier parameterisation may be used in those spatio-temporal regime-switching studies that have been carried out using discrete Markov switching [11]. Heavy parameterisation may lead to improved in-sample fit but does not necessarily produce improved out-of-sample predictions such as short-term infection forecasts [48].

Spatio-temporal infection data also raise the issue of neighbourhood infection spillover, which is not included in some spatio-temporal infection models, e.g., [11,43]. In the analysis above, we introduce regime alternation into an autoregressive space–time framework to reflect pronounced fluctuations in infection levels, while also allowing for neighbourhood spillover in infection, which is itself governed by regime alternation.

We show how the proposed method provides improved fit and short-term predictions compared to other spatio-temporal infection models that do include infection spillover but have no adaptation to epidemic phasing [24,25]. Detailed results from the two case studies, which have different variants involved in epidemic peaks and different spatial frameworks, confirm the utility of the model.

In the near future, recurrent epidemic phases of COVID may occur as a result of new variants even if the disease takes on endemic features. In such a situation, appropriate modelling techniques provide relevant research contributions to monitoring and containing the impacts of COVID and the above paper is intended as one such contribution.

The case studies in this paper both concern COVID, whereas other regime-switching applications include dengue [9] and influenza [11]. Hence, a full evaluation of the method here should include application to other infectious diseases with evaluation including out-of-sample predictions.

## Figures and Tables

**Table 1:** Comparative Model Fit, London Boroughs (*N* = 32, *T* = 96).

Fit to Observed Data (*T* = 95)							
		WAIC		RPS		DSS	
Model 1		32,267		185,300		24,683	
Model 2		31,689		180,500		23,930	
Model 3		29,292		111,024		22,467	
Model 4		29,727		111,345		23,177	
**One-Step Ahead Prediction, Actual Count: 155,181**							
**95% Interval for Y_T+1_**							**RPS_T+1_**
	**Mean**		**2.5%**		**97.5%**		
Model 1	193,600		168,700		224,800		96,920
Model 2	192,600		168,300		224,300		95,520
Model 3	162,897		154,613		171,439		40,823
Model 4	163,584		154,018		175,048		42,923

**Table 2:** Weeks with Highest Growth in Cases, Total Areas (from 32) with $\rho_{it}$ > 1.

Week (t)	Posterior Mean Total Areas	2.5%	97.5%
94	32	32	32
2	32	32	32
3	32	30	32
42	32	30	32
41	30	26	32
4	30	25	32
30	27	19	32
69	27	18	32
31	25	16	32
72	22	11	30
27	21	9	31
70	20	10	29
93	20	9	29
66	14	1	30
65	4	0	15
67	3	0	12
32	3	0	9
34	2	0	7
24	2	0	10
71	1	0	4

**Table 3:** Comparative Model Fit, Southeast England (*N* = 144, *T* = 21).

Fit to Observed Data (*T* = 20)							
		WAIC		RPS		DSS	
Model 1		27,579		118,685		22,317	
Model 2		27,613		118,148		22,384	
Model 3		25,439		65,663		19,348	
Model 4		25,346		65,092		19,145	
**One-Step Ahead Prediction, Actual Count: 210,099**							
**95% Interval for Y_T+1_**							**RPS_T+1_**
	**Mean**		**2.5%**		**97.5%**		
Model 1	195,244		181561		210,088		27,025
Model 2	195,545		181644		210,548		27,223
Model 3	202,268		192260		213,112		24,062
Model 4	204,026		194169		214,482		23,661

**Table 4:** Ranked Probability Score (RPS) (Posterior Means by Week and Model).

Week	M1	M2	M3	M4	Total Cases, Greater Southeast, Yt	Relative Increase in Cases Compared to Previous Week	Ratio of RPS M1 to M4
2	541	536	423	429	2161	1.23	1.26
3	655	657	508	513	2641	1.22	1.28
4	848	851	669	678	3972	1.50	1.25
5	914	917	692	698	4458	1.12	1.31
6	1379	1367	637	637	4030	0.90	2.16
7	1469	1471	1060	1044	6846	1.70	1.41
8	2135	2138	1608	1527	11,365	1.66	1.40
9	2464	2474	1770	1723	17,034	1.50	1.43
10	2453	2462	1826	1823	20,523	1.20	1.35
11	3955	3976	2591	2554	29,633	1.44	1.55
12	5140	5077	2939	2921	30,263	1.02	1.76
13	4566	4564	3307	3279	34,546	1.14	1.39
14	5327	5319	3649	3676	45,007	1.30	1.45
15	10,898	10,875	3729	3412	38,227	0.85	3.19
16	8410	8374	3311	3027	34,345	0.90	2.78
17	4976	4992	3737	3615	41,090	1.20	1.38
18	11,534	11,427	5598	5693	67,090	1.63	2.03
19	31,660	31,474	10,671	10,405	127,905	1.91	3.04
20	19,360	19,197	16,937	17,439	154,518	1.21	1.11

## Data Availability

Data obtainable at https://coronavirus.data.gov.uk/ (accessed on 3 May 2022).

## Supplementary Materials

The following supporting information can be downloaded at: https://www.mdpi.com/article/10.3390/ijerph19116669/s1, File S1: Code and Spatial Data; File S2: Incidence Data Greater South East; File S3: Incidence Data London Boroughs.
